# Recruitment and retention of occupational therapists and physiotherapists in rural regions: a meta-synthesis

**DOI:** 10.1186/1472-6963-13-59

**Published:** 2013-02-12

**Authors:** Robin K Roots, Linda C Li

**Affiliations:** 1 Department of Physical Therapy, University of British Columbia, Vancouver, BC, V6T 1Z3, Canada; 2 Milan Ilich Arthritis Research Centre, 5591 No. 3 Road, Richmond, BC, V6X 2C7, Canada

**Keywords:** Recruitment, Retention, Workforce, Physical therapy, Occupational therapy, Continuing professional development

## Abstract

**Background:**

Significant efforts have been made to address the shortage of health professionals in rural communities. In the face of increasing demand for rehabilitation services, strategies for recruiting and retaining occupational therapists (OTs) and physiotherapists (PTs) have yielded limited success. This study aims to broaden the understanding of factors associated with recruitment and retention of OTs and PTs in rural regions, through a synthesis of evidence from qualitative studies found in the literature.

**Methods:**

A systematic search of three databases was conducted for studies published between 1980 - 2009 specific to the recruitment and retention of OTs and PTs to rural areas. Studies deemed eligible were appraised using the McMaster Critical Review Form. Employing an iterative process, we conducted a thematic analysis of studies and developed second order interpretations to gain new insight into factors that influence rural recruitment and retention.

**Results:**

Of the 615 articles retrieved, 12 qualitative studies met the eligibility criteria. Our synthesis revealed that therapists’ decision to locate, stay or leave rural communities was influenced to a greater degree by the availability of and access to practice supports, opportunities for professional growth and understanding the context of rural practice, than by location. The second-order analysis revealed the benefits of a strength-based inquiry in determining recruitment and retention factors. The themes that emerged were 1) support from the organization influences retention, 2) with support, challenges can become rewards and assets, and 3) an understanding of the challenges associated with rural practice prior to arrival influences retention.

**Conclusions:**

This meta-synthesis illustrates how universally important practice supports are in the recruitment and retention of rehabilitation professionals in rural practice. While not unique to rural practice, the findings of this synthesis provide employers and health service planners with information necessary to make evidence-informed decisions regarding recruitment and retention to improve availability of health services for rural residents.

## Background

Rural health care is gaining recognition as a distinct entity with unique challenges. In Canada, rural health is characterized by a higher prevalence of chronic diseases and traumatic injuries [1] as compared with urban areas, as well as higher rates of overweight and obesity [2], lower life expectancy [1], and fewer health care resources including health professionals [1,3]. While no single definition of rural exists, Statistics Canada defines ‘rural and small town’ as regions that have a population of less than 10,000 [2]. According to the 2006 Census, approximately 20% of the Canadian population live in these regions [4]. The relatively poorer health profile in rural communities, combined with an aging population, suggest that there is a proportionally larger demand for services for medical treatment, rehabilitation, and health promotion.

Occupational therapists (OTs) and physiotherapists (PTs) are critical members of the health care team, providing direct patient care, education, and advocacy in the community. Despite the high demand for services [5], only 5.9% of the occupational therapy workforce and 7.7% of the physiotherapy workforce in Canada worked in rural areas in 2010 [6,7]. Furthermore, the trend of migration from urban to rural is steadily decreasing since 1996 when it was from 9% for PTs and 8% for OTs, to 2% for PTs and a reverse of 2% OTs migrating from rural to urban in 2001 [8]. The imbalance in the supply and demand for health care services in rural and remote regions results in inequitable access to services [9].

To address the disparity between the need for and supply of health services, there has been a growth in research on recruitment and retention of health care workers in rural and remote areas. Despite the wealth of studies in this field, the shortage of health care professionals from all disciplines in rural communities does not seem to have abated signalling the need for a better understanding of the strategies necessary to improve the situation.

Strategies, such as educational interventions [10], financial incentives, regulations, and practice support interventions [11], have been used to attract health professionals to work in rural and underserviced areas. However, the effectiveness of these measures has not been well established in the literature. A 2009 Cochrane review of these interventions by Grobler et al. concluded the evidence to be very weak due in part to poor study designs [12]. The majority of studies fail to measure the effectiveness of the strategies, resulting in frequent repeats of similar programs with limited progress in the field. In an analysis of the determinants of geographical maldistribution of the health workforce, Dussault and Franceschini noted that most of the strategies targeted short-term factors, such as individual and social factors [13], and neglected to address broader factors, such as organizational challenges, which have a much longer lasting effect. Wilson et al. reviewed rural workforce policy in Australia and noted a focus on the shortage of physicians rather than the shortage of all health professionals across the health care system [14]. Lee and Winters also found that recruitment and retention strategies centred on medicine and nursing [15].

Given the differences in the contextual environment in which professionals practice, recruitment and retention strategies from medicine and nursing may not be transferable to rehabilitation professionals. For example, OTs and PTs often work in isolation [10], which may result in different individual, social and organizational needs. These differences further challenge health human resource workforce planning and policy [14]. Hence, in order to improve access to health care services for people living in rural areas, we need to better understand key contextual factors in recruiting and retaining health providers to rural areas, in particular OTs and PTs [16,17].

### Factors specific to the rehabilitation workforce

Research on the determinants of individual factors associated with recruitment of rehabilitation professionals to rural areas has identified a strong association with having a rural background [18,19], exposure to rural practice during training [18], and financial incentives such as loan forgiveness [19]. Personal characteristics associated with recruitment and retention to rural areas include desire to serve community needs [19] and perceived satisfaction with opportunities for professional growth [10,20]. Social factors that are common to both recruitment and retention of OTs and PTs include proximity to family [10], desire for a rural lifestyle [10,19], attractive job opportunities for spouse [10], and career and family ties [21].

Studies on retention of OTs and PTs have centred on understanding factors associated with relocation to larger centres. Individuals who leave their rural practice tended to be younger, male and single [20], lived far away from their family, reported a lower spousal satisfaction with rural lifestyle, and reported poor job opportunities for self and spouse [21]. Organizational factors, such as management structure and lack of professional support, were also associated with OT and PT attrition [21]. In a study by Beggs and Noh, PTs in Northern Ontario were more likely to remain in rural areas if they were in private practice [20]. The authors suggested that this might be due to the autonomy afforded to private practitioners, seen as important to the provision of services in rural areas.

While observational studies have provided ample factors associated with recruitment and retention of health professionals in rural areas, there has been limited success in mitigating the maldistribution of the health human resource workforce. Dussault and Franceschini concluded that the multifaceted nature of recruitment and retention requires a multifaceted strategy [13] and understanding of the context in which practitioners’ practice. To our knowledge, there has not been a review of the qualitative literature on recruitment and retention of rehabilitation professionals in rural areas to examine the context, such that lessons learned may be integrated into multifaceted strategies to increase the number of OTs and PTs in rural areas.

To broaden our understanding of why some factors and determinants are important to recruitment and retention, and the influence that context has on the recruitment and retention of OTs and PTs to rural areas, we conducted a meta-synthesis of the qualitative literature in this area. A meta-synthesis is designed to advance knowledge through a summary of the qualitative research beyond what is already known [22]. This methodology helps to move policy forward through enlarging our understanding [23] and offering direction for future research.

## Methods

### Search strategy and article review

A systematic literature search was conducted by one of the authors (RKR) using Medline (1980 to December 2009), EmBase (1980 to December 2009) and CINAHL (1982 to December 2009). Search terms used included: ‘rehabilitation’, ‘rehabilitation professionals’, ‘allied health professions/ professionals / personnel / occupations’, ‘physiotherap*’, ‘occupational therap*’, ‘rural’, ‘rural health’, ‘rural service delivery’, ‘health care services’, ‘workforce’, ‘recruitment’ and ‘retention’. In addition, we performed two hand searches. The first was of reference lists of all articles selected as meeting our inclusion criteria. The second hand search involved journals selected as relevant and pertinent to this area: *Australian Journal of Rural Health* and the *International Electronic Journal of Rural and Remote Health Education, Research and Policy* published between 2005 and 2009.

Articles were eligible for the review if they met the following criteria: 1) included OT and PT research participants; 2) reported on issues, factors, and/or strategies related to recruitment and retention; 3) focused on rural and/or remote areas; and 4) used qualitative methodologies or analytical techniques. As no standardized definition of rural exists internationally in the literature, we accepted all definitions of rural. Articles were excluded if they did not use qualitative methodologies or analytical techniques, or if they were not published in English. Results of the search were compiled and the first author (RKR) screened all titles and abstracts and reviewed the full content of articles that met the inclusion criteria.

### Data synthesis

We followed the methodological framework for a meta-synthesis outlined by Gewurtz et al. [22]. While appraisal of the qualitative literature remains a controversial topic due to its origins in the quantitative paradigm [24-28], we chose to assess the quality of all eligible articles to advance understanding of the context of each study and the variations in the qualitative literature. The level of quality of articles was not a criterion of exclusion [29,30], however we hope that it serves to inform future research. We used the Critical Review Guidelines for Qualitative Studies developed by the McMaster University Occupational Therapy Evidence-Based Research Group [31] as it aligned most closely with the nature of the literature being reviewed. Each article was evaluated for quality based on eight criteria: 1) study purpose; 2) literature; 3) study design; 4) sampling; 5) data collection; 6) analyses; 7) rigour; and 8) conclusions / implications. The scale (Table 1) is detailed in its evaluation of the research quality and can be scored on a point system (each section receiving a score of 1 = the criterion was met; 0 = the criterion was not satisfied), with a maximum score of 18 [32,33].

**Table 1:** Appraisal of the literature using critical review guidelines for qualitative studies developed by the McMaster University occupational therapy evidence-based research group

	**Purpose**	**Literature review**	**Design**	**Theory**	**Method**	**Sampling**	**Saturation**	**Consent**	**Descriptive clarity**	**Procedural rigour**	**Analytic rigour- inductive**	**Findings consistent**	**Audit trail**	**Process of analysis**	**Conceptual framework**	**Overall rigour**	**Conclusion**	**Contribution to theory**	**Overall score / 18**
**Bent 1999**	Y	Y	Y Descriptive	N	Y Interviews	N	N	N	Y	N	Y	Y	N	Y Content analysis	N	N	Y	N	9
**Butler & Sheppard 1999**	Y	Y	Y Mixed methods comparative	N	Y Questionnaire	Y	Y	Y	Y	Y	Y	Y	N	Y conceptualization, cataloguing, linking	Y	Y	Y	Y	16
**Mills & Millsteed 2002**	Y	Y	Y Ethnographic	Y	Y Interviews	Y	N	Y	Y	Y	Y	Y	Y	Y Content analysis	Y	Y	Y	Y	117
**Lee & MacKenzie 2003**	Y	Y	Y Exploratory	N	Y Interviews	Y	N	Y	Y	Y	Y	Y	Y	Y Coding, comparative	N	Y	Y	N	14
**Denman & Shaddock 2004**	Y	N	Y Mixed methods	N	Y Focus groups, interviews & surveys	N	N	N	N	N	N	Y	N	N	N	N	Y	N	5
**Steenbergen & MacKenzie 2004**	Y	Y	Y Descriptive	N	Y Interviews	Y	N	Y	N	N	N	Y	N	Y Codes & themes	N	N	Y	N	8
**Devine 2006**	Y	Y	Y Phenomenology	Y	Y Interviews	Y	Y	Y	Y	Y	Y	Y	Y	Y Thematic analysis	N	Y	Y	Y	17
**Gillham et al 2007**	Y	Y	Y Descriptive	Y	Y Interviews	Y	Y	Y	Y	Y	Y	Y	N	Y Thematic analysis	N	Y	Y	Y	16
**Thomas & Clarke 2007**	Y	Y	Y Narrative inquiry	Y	Y Focus groups	Y	N	Y	Y	N	N	Y	N	N Codes	N	N	Y	Y	11
**Boshoff & Hartshorne 2008**	Y	Y	Y Descriptive	N	Y Questionnaire	N	N	N	Y	N	N	Y	N	Y Descriptive statistics Content analysis	Y	N	Y	N	9
**Le & Kilpatrick 2008**	Y	Y	Y Exploratory	N	Y Interviews & written statement	N	N	Y	Y	Y	Y	Y	N	Y Thematic analysis	Y	Y	Y	N	13
**Manahan et al 2009**	Y	Y	Y Descriptive	Y	Y Interviews	Y	Y	Y	Y	Y	Y	Y	Y	Y Thematic content analysis	Y	Y	Y	Y	18

This meta-synthesis was conducted in accordance with the methodologies outlined by Mays and Pope [26], and the procedures used by Reid et al. [32] and Sandelowski and Barroso [23]. Initially, all factors relating to recruitment and retention were extracted. Data synthesis was completed by the first author (RKR) and reviewed by the second author (LCL) to ensure congruency of themes. Thematic analysis [34] was chosen as the method of analysis as it allowed us to examine why certain factors and determinants were important to recruitment and retention of OTs and PTs to rural areas, as well as to identify the influence of context on the recruitment and retention.

Each eligible article was reviewed, the recruitment and retention factors were identified (Table 2), and the contextual foundations of the research were extracted (Table 3). The findings were summarized and inductively analysed for common and recurring themes [35]. Similarities and variations were juxtaposed and translated from one study to another [26] and consideration was given as to how each theme related to each study. Through this process, new explanations for why and how themes occurred were identified and re-evaluated in the context of the literature. This integrated methodology of inductive and deductive thematic analysis resulted in second order conceptual themes. Finally, we considered these concepts for policy and practice implications.

**Table 2:** Characteristics of included studies

	**Context / Setting**	**Definition of rural**	**Sample**	**Characteristics of rural practice / rural practitioners**
**Bent 1999**	Central Australia	Central Australia, Northern Territory considered to be remote	17 OTs, PTs, SLP; excluded private practice, management or consultants	Large clinical caseloads, large geographical area, variety of age groups and conditions
**Butler & Sheppard 1999**	Australia	Rural defined as < 25000	58 PTs graduated within 2 years; (18 in rural and 40 in metropolitan areas)	More likely to be sole charge, less professional support, greater role as educator
**Mills & Millsteed 2002**	Australia	Broad definitions cited and used	10 OTs previously in rural practice; purposive sample and snowball	Breadth and depth of professional knowledge gained useful in all areas/settings of practice
**Lee & MacKenzie 2003**	New South Wales, Australia	Classification based on density and distance	5 OT new graduates, (4 in public practice and 1 in private)	Varied caseload, limited resources, limited support, greater interactions with clients and integration into community, professionals require independence & self-confidence
**Denman & Shaddock 2004**	New South Wales, Australia	Work location > 1 hour drive from a metropolitan region (<250,000)	Focus group of 1 OT, 2,PT, 2 SLP; 31, surveys returned from 9 OTs, 7 PTs, 13 SLP. Interviews with 1 SLP, 2 mangers working in departments providing disabilities services	None given
**Steenbergen & MacKenzie 2004**	New South Wales, Australia	Participants decided	9 OTs in 1^st^ year practice in rural; (7 public and 2 private sector)	None given
**Devine 2006**	Australia	Australian accessibility remote index	6 OTs newly graduated; 4 OT instructors	Greater need for management skills, prioritization, time management
**Gillham et al 2007**	Victoria, Australia	Classification based on density and distance	8 allied health profession students, 7 managers, 18 allied health professionals and 10 former staff; all public sector	None given
**Thomas & Clarke 2007**	Northern Territory, Australia	None given	18 AHP including OTs and PTs	Skills and attributes for rural practice: being organized, creative, flexible, cooperative and collaborative, cultural awareness, communication, resourceful, reflective learner, networking, dual roles and responsibility
**Boshoff & Hartshorne 2008**	South Australia, Australia	Combined use of terms country and rural, no definitions	18 OT managers completed Questionnaire; majority public sector	Multi-skilling of therapists, problem solving,
**Le & Kilpatrick 2008**	Australia	None given	6 overseas born Australian trained health care professionals including 1 PT	None given
**Manahan et al 2009**	British Columbia, Canada	Broad definitions cited and used	6 AHP including: 6 SLP, 4 OTs, 4 PTs; no indication whether public or private sector	Variety, change, dual relationships, challenges, need for creativity

**Table 3:** Meta-synthesis of recruitment and retention factors

	**Recruit factors**	**Retention factors**	**Recruitment and retention strategies**
**Bent 1999**	Working with aboriginal populations, team environment, permanent position, diversity of work, bush travel Deterrents: insufficient staff, management and organizational problems, professional isolation, vacant positions results in increased work	Access to professional development, understanding of rural practice skills by management and support from management, cost of living, networking and communication with peers	Cross-cultural training / education, professional networking, management support, information technology, heightened profile of rural practice- focus on the positives of living/ working in rural, acknowledgement of rural practice as a specialty
**Butler & Sheppard 1999**	Rural childhood and final placement in rural (regardless of mandatory) Students felt prepared to take up positions in rural areas Rural training module did increase interest in working in a rural area and awareness of life in rural community	Retention factors not identified Prior background in rural inconclusive Similar levels of support noted for rural and metro graduates	Support and professional development critical for new graduates regardless of geographical location
**Mills & Millsteed 2002**	Appeal of the opportunity, variety of tasks Deterrents: lack of orientation to position and to community, lack of support, isolation, high workload	Personal factors- social sphere, compensation Professional factors-rural practice issues, rural experience Retention is the balance between incentive to stay and incentives to leave	Orientation and assistance with building contacts
**Lee & MacKenzie 2003**	Rural lifestyle, rural background, clinical opportunity Deterrents: perceived lack of support	Rural placement reinforces intentions to stay in rural community; professional support valued	Recruitment from rural background and rural fieldwork experience. Peer support, feedback and evaluation, social contact and support networks
**Denman & Shaddock 2004**	Lifestyle and personal factors, evidence of support for professional development, ‘critical mass’ of staff, professional supervision, career structure	Reasons to leave: partner moves away, resource limitations, insufficient support, opportunity for professional development, vacancies, work not valued by management	Flexibility by management (accommodation for concerns relating to why people leave or stay), creation of cross-sectorial teams, remuneration and employment conditions individualized
**Steenbergen & MacKenzie 2004**	Professional support to build professional identity	Wide variety of resources required Reasons to leave depleted resources and decreased education	New graduates require support and professional development to establish professional identity regardless of geographical location
**Devine 2006**	Rural background (self or spouse), attracted to position not location, multidisciplinary team, personal autonomy, development of skill Undergraduate program focused on problem solving skills and ability to work autonomously	Professional support including educational preparation and development Clinical placement in rural setting did not adequately prepare for rural practice	Preparation by undergraduate course (including subjects such as health promotion and primary health care, autonomy) Development of skills and support for competency throughout career
**Gillham et al 2007**	Rural background, rural placement or rural connection, career opportunities (rotating positions for new graduates), financial incentives, career progression, mentoring, access to professional support, supervision, social networks (especially for students)	Career/ professional development, social network opportunities, management style & organizational policy (collaboration between staff & management, consultation & open communication), flexibility in work schedule, financial remuneration for accommodation & relocation	Mentors, adequate human resources in order to best practice, career opportunities
**Thomas & Clarke 2007**	Knowledge of community, role of health care professional in community	Relationships with other professionals and community; time management, responsibility in the community, personal resourcefulness, adventure Reasons to leave: stress, responsibility	Development of skills and attributes in training programs
**Boshoff & Hartshorne 2008**	Variety of caseload and services delivered	Reasons to leave: limited resources, high client to therapist ratio, lack of professional support, strategies and collaboration	Service delivery model specific to the setting, networking and collaboration to reduce isolation, workplace support, account for recruitment factors and profile of practice
**Le & Kilpatrick 2008**	Autonomy, the family oriented nature of rural communities	Reasons to leave: cultural shock, lack of social and emotional support, communication and lack of collegiality	Professional development, assistance in accommodating cultural needs and decreasing distance through connections with colleagues
**Manahan et al 2009**	Rural background, availability and accessibility of training programs in rural areas determined career choice, decision to work in rural prior to work, need for health care professionals in rural community, positive past experience in rural community	Age and stage of life, proximity to family, career advancement opportunities, peer support, affordability, lifestyle, congruent with life values	Account for age and stage of life, values in addressing personal and professional factors, admissions selection criteria of training programs

## Results

### Systematic search

**Figure 1:**
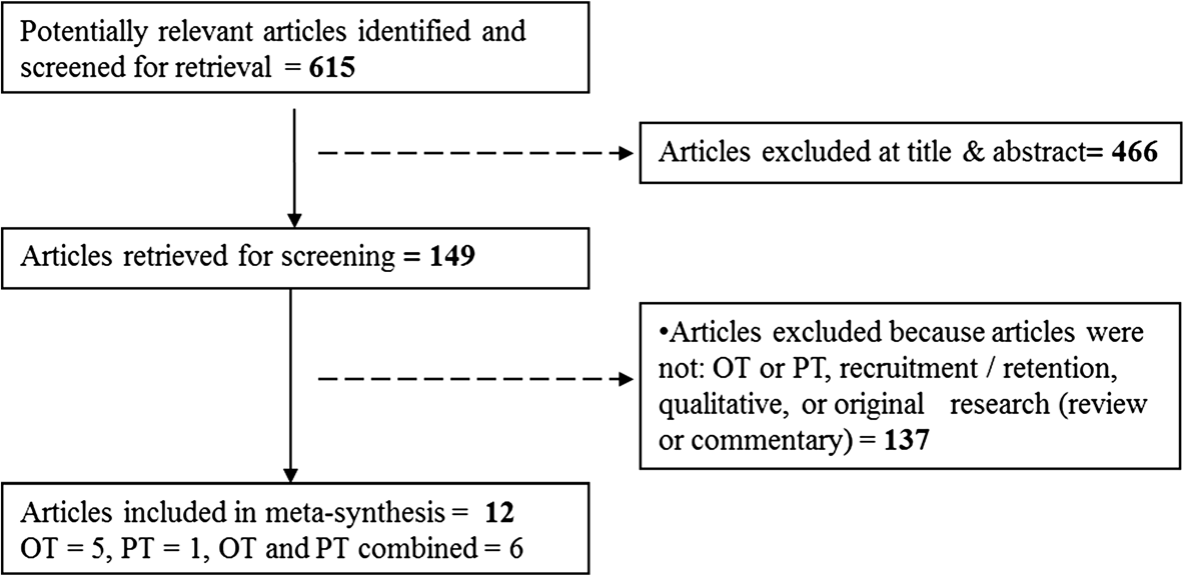
Literature search results.

The systematic literature search retrieved 615 articles, 12 of which met the eligibility criteria (Figure 1). With the exception of one study set in northern British Columbia [36], the research included in this synthesis originated from Australia where the issue of health human resource shortages in rural areas is a focus of much rural health research.

While all studies reviewed in this synthesis included OTs and PTs, there was significant variation in the sample populations (Table 2). Five of the studies included students or new graduates [37-41], and two studies included therapists who no longer worked in rural or remote areas [39,42]. Across studies, there was no consistency in the definitions of rural (Table 2). This further increases the complexity of comparing findings across the literature and applying conclusions to rural practice contexts.

### Methodological appraisal

Each study was evaluated for elements of high quality qualitative research (Table 1) and to consider the impact that the quality and rigour of each study had on the overall findings. The majority of studies had a total score greater than 10 out of 18 and included the elements of a clearly stated purpose, appropriateness of design, methods and sampling, consent and findings consistent with the data collection. The weakest areas amongst the studies synthesized were details of sampling procedures, use of theory, conceptual frameworks, contribution to theory and overall rigour. The general absence of theory and conceptual frameworks makes transferability of results from these studies difficult [43].

Of the studies reviewed, two indicated that they used a descriptive methodological approach, two employed an exploratory approach, one explicitly used an ethnographic methodological approach, one study applied a phenomenological approach, and one used a narrative approach. The remaining articles failed to indicate the qualitative methodological approach used and the majority did not ascribe to any theory to guide their research. Thematic analysis was used by five studies and content analysis was used by two studies. One study noted the use of conceptualization and another conducted coding, but did not describe the process or categorization. Three studies did not provide details of the analytic process. All studies conducted interviews and/or focus groups, and some added a questionnaire or survey. Only two of the 12 studies described triangulation of data, both of which used a mixed methods design [37,44]. Member checking and use of an external auditor were used to ensure research rigour in two of the 12 studies [36,40].

### First order analysis - themes

Our first order analysis found organizational support [39,41,42,44,45] and opportunities for professional development [41,46] to be important factors in attracting and retaining OTs and PTs in rural areas. On first glance, these factors did not appear to be unique to rural practice as they are universally valued by health professionals regardless of geographical location. However, when organizational support and professional development were viewed in light of the rural context and the challenges associated with accessing professional development when practising in a rural area, these factors appeared to have a stronger influence on recruitment and retention than the common factors of geographical location relative to family and personal choices [45,46]. Three themes emerged in the first order analysis: 1) the availability of professional support was important to attracting and retaining PTs and OTs in rural areas; 2) the importance of opportunities for professional growth; and 3) the nature of rural practice.

### Theme 1: the availability of professional support was important to recruitment and retention

Professional support was cited by all 12 studies as important to OTs and PTs working in rural areas. Our analysis identified the availability of professional support for rehabilitation professionals in rural areas as a key factor for recruitment and retention. Professional support included: 1) an understanding of the characteristics of rural rehabilitation practice by managers, the profession and other team members, and 2) having adequate resources to fulfill the requirements of the position/practice and access to continuing professional development.

In the analysis, we noticed that OT and PTs’ perception of the extent to which support was available varied enormously. Some therapists felt supported by management [39,47] and by colleagues in other professions [39], whereas others perceived a general lack of support by management and by urban practitioners in their own profession [40,42,45]. Receiving adequate professional support was considered dependent on the managerial style and/or the overarching organizational policy [39,44,45,47,48]. OTs and PTs described feeling supported when management offered them flexibility in their work schedule to accommodate distance travelled [39], or recognized the increased workload that occurred due to the shortage of rehabilitation staff [45]. Perceived lack of professional support appeared to result in poor retention [45], or the reluctance to accept or stay in a position [38,40].

Professional support was cited as particularly important for new graduates in rural areas to assist them to develop their professional identity [40] and transition from student to professional [37-39,44]. In urban and suburban centres, this could be achieved through contact with and mentoring of peers [37,40]. However, because OTs and PTs in rural settings often work in isolation, access to this type of informal professional support was limited and posed a barrier to recruitment [38,42]. In a comparative study, Butler and Sheppard noted that rural therapists were less likely to have access to professional support than metropolitan therapists [37]. Half of the participants in a study by Manahan et al. noted the importance of peer support in their decision to stay in rural practice [36].

Transcending the challenge of obtaining professional development in rural areas required networking, collaborating, problem-solving, shadowing, and mentoring other health care professionals [36,38,42,44,47,49] and management [39]. Working closely with colleagues provided support to OTs and PTs unfamiliar with procedures, and an opportunity to communicate regularly and build trusted relationships [44,49]. Denham et al. noted that participants felt having a critical mass of staff was essential for reducing the sense of isolation [44]. Interestingly, some therapists perceived managers from a different discipline as lacking the understanding of the types of services offered by rehabilitation professionals [45]. This appeared to lead to a sense of decreased professional support [45].

Our analysis also suggested that limited resources in rural areas were a challenge to practice [38,44]. While some studies did not distinguish between human resources and physical resources [40], the setting in which participants worked also appeared to be important. More participants who worked in the public sector identified having limited resources [40,44] or not being adequately resourced [41,42,45] than did participants in private practice [41]. Gillham and Ristevski suggested that the flexibility and autonomy of private practice might have contributed to a perception of greater control over the issues around resources [39]. It should be noted that two studies [42,45] excluded participants if they worked in private practice. With the exception of a study by Boshoff et al. that surveyed front-line OTs and PTs as well as managers [47], therapists in a managerial role did not perceive the additional need for support that resulted from geographical or professional isolation.

In summary, while professional support was found to be a key factor for rural recruitment and retention, it was apparent through the analysis that this support needed to come from management and/or organizations that understood rural practice, and from managers of a rehabilitation discipline.

### Theme 2: importance of opportunity for professional growth

Access to and availability of professional development were listed as the greatest challenges to practising in a rural area [36,38,39,41,45,47]. Having ‘room to grow professionally’ [42] and ‘opportunity to grow’ within their career [36] appeared to be a central factor [39] in the recruitment and retention of rehabilitation professionals in rural areas.

In the studies reviewed, OTs and PTs recognized that the challenges of rural practice, such as the diversity of the caseload, offered the opportunity to build their skills in a number of different areas [47], and to be ‘expert generalists’ in rural practice [45]. The breadth of rural practice was noted by some participants [38,39] as a good starting point for their career as it allowed them to consider what areas they would later specialize in [42]. Nevertheless, participants in a study by Butler and Sheppard felt they needed more experience before taking on a rural position [37]. Regardless, rural employment offered greater responsibility and personal autonomy [38].

Participants in a number of studies noted that insufficient room to grow professionally or a flat career structure, combined with limited professional development opportunities, contributed to decreased job satisfaction [45] and workforce attrition [38,39,44]. Lee and McKenzie also found fear of deskilling to be a reason for practitioners’ reluctance to go to or stay in a rural area [40].

### Theme 3: understanding the nature of rural practice

An understanding of the nature of rural practice played a vital role in recruiting and retaining rehabilitation professionals. Across studies, rehabilitation practice in rural areas was described as occurring in a variety of workplace settings with a diverse caseload [45], a high client to therapist ratio [47], and the necessity of therapists to have a wide set of clinical and professional skills [38,47]. As compared with OTs and PTs working in urban areas, therapists in rural areas were found to have a larger role in providing education, greater one-on-one time with clients [37], and a greater proportion of work time travelling [47]. Devine et al. noted that it was the professional opportunities associated with rural practice that attracted therapists to the position rather than the geographical location [38]. For example, in a study by Bent, the opportunity to work with aboriginal populations was identified as a feature of rural practice that brought rehabilitation professionals to rural areas [45]. Knowing and understanding these features of practice appears to have an impact on recruitment and retention. Butler and Sheppard suggested that clinical placements in rural areas did not increase rural recruitment; however, placements contributed to retention due to more informed decision making regarding location of practice [37].

A number of studies in this review suggest that OTs and PTs who participated in these studies felt that their colleagues, other health care professionals or organizations did not understand the nature of rural practice, or recognize the distinct features of rural practice [38,39,42,45]. It is generally accepted that this lack of understanding and recognition contributes to poor recruitment and retention. Our first order analysis highlighted the importance of professional support, opportunity for professional development and understanding the features and context of rural practice. This resulted in deeper analysis to produce second order themes and the development of new interpretations of features of recruitment and retention of OTs and PTs in rural areas.

### Second order analysis

Through deeper analysis of each theme in the first order, we gained additional insight into factors that contribute to the recruitment and retention of OTs and PTs to rural areas. When we unpacked the first theme (availability of professional support) for greater understanding, it became evident that professional support from the organization was critical to retention. Embedded in the second theme of professional growth was the transformation of practice challenges into rewards and assets, which also contributed to retention rather than recruitment. Finally, analysis on the importance of understanding rural practice illustrated that understanding prior to practising in a rural area was a key factor for recruitment and retention of OTs and PTs to rural practice.

### Professional support from the organization influenced retention

Professional support was cited as important to OTs and PTs working in rural areas irrespective of the stage of career. However, when we examined the factor of professional support more closely, we noted that its influence on recruitment or retention could be delineated along stages of career. Five studies [37-41] included new graduates in their sample and associated availability of professional support with recruitment to rural practice. In a comparison of new graduates in rural areas to those in urban areas, Butler and Sheppard found negligible difference in participants’ perception of support for practice [37]. This suggests that new graduates seek support to develop a professional identity and obtain a comfort level with their professional skills regardless of practice location.

Lack of professional support by the organization was cited commonly as a reason for leaving rural practice. Thus, it would appear that promises of support such as continuing education, adequate orientation and managerial support, which acted as attractants to rural practice, did not materialize in the rural settings and contributed to attrition. The perception by OTs and PTs of professional support being provided by the organization was a significant factor in retention of rehabilitation professionals to rural areas.

### With support, challenges can become rewards and assets

Several studies employed an appreciative approach in their research design, which reflected the positive perspective by which some OTs and PTs viewed the isolation and resource scarcity of rural practice, and shed a positive light on the challenges presented in rural practice [40,42,45]. Furthermore, appreciation shown by a community for rehabilitation services contributed to job satisfaction and retention [40].

Initial analysis led to confusion as to whether a particular factor contributed to recruitment or retention. Factors that attracted therapists to rural practice appeared to be the same as those that deterred therapists from entering or remaining in rural practice [42,49]. For example, the rural setting may have initially been an attractant but geographical isolation may also contribute to attrition [42]. It appeared that some OTs and PTs sought the challenge of rural practice but also left because of the challenge. When asked what the challenges and rewards were, participants noted autonomy [38,49], diversity of practice [45,47], relationships [46], and the need for broader skills [38]. However, it was not clear whether the challenges faced by OTs and PTs were attributed to the position that they were in or to being in a rural location [40]. Studies using a strength-based line of inquiry in research framed these features as positive factors to recruitment and retention necessitating a closer look at research study design.

Converting the challenges of rural practice into rewards through positive experiences appeared to be associated with retention. For example, if a therapist chose rural practice based on a desire for autonomy or working with an aboriginal clientele and they received the support necessary to remain in that role, this challenge would be considered a positive reason to stay in rural practice [45]. Conversely, if a therapist was not prepared for the autonomy of rural practice, or did not receive adequate support by management to maintain or build the knowledge needed for providing culturally appropriate services, the challenge resulted in attrition [45].

When observing the results of the second theme, the importance of opportunities to ‘to grow’ professionally was linked with job satisfaction and was a significant predictor of retention. Through interviews with therapists who left rural practice, Mills and Millsteed found that those who had the opportunity for advancement while in rural practice stayed the longest [42]. The opportunity for advancement was a consistent factor in retention across all stages of career [36,39,41,42].

### An understanding of the nature of rural practice prior to arrival influences retention

In examining the third theme, it became clear that prior understanding of the nature of rural practice had a significant effect on decisions to leave or to stay in rural. Whether obtained through lived experience or through clinical education fieldwork placements, an understanding of the nature of practice in a rural setting prior to obtaining the position appeared to affect retention. A few studies [36,40,49] identified that OTs and PTs who had made the decision to pursue rural practice prior to being offered a position recognized the rewards in rural settings more so than the challenges.

The mixed messages in the literature regarding the role of clinical education in influencing students and graduates to work in rural areas [37,38] suggest that training programs do not consistently assist students in understanding the nature of rural practice. Boshoff and Hartshorne found that the majority of recently graduated OTs did not feel adequately prepared for rural practice [47], but it was not clear whether these students had participated in rural placements or were familiar with rural practice prior to working in a rural area. In contrast, in 1999 Butler and Sheppard found that newly graduated PTs were equally well prepared or better prepared in rural practice as compared to metropolitan practice [37].

Finally, while the analysis showed that prior understanding of the nature of rural practice was critical to retention, it remained unclear whether clinical education in rural areas had a direct effect on recruitment or retention.

## Discussion

Findings from this meta-synthesis suggest that professional support from management and organizations is critical in the decision making of OTs and PTs to work in rural practice. Support was demonstrated through recognition and understanding of the features of rural practice, such as larger caseloads [40,45,50], limited referral options [50], decreased access to resources [37,40,46,50] and limited access to continuing education [37,39,45,50], and through providing opportunities to grow professionally and convert these challenges into opportunities for a rewarding career.

On close examination of these first order themes during the second order analysis, we found that professional support and prior understanding of rural practice had a greater influence on retention than recruitment. Our second order analysis also found that the line of inquiry of a study has the potential to influence whether the factor has a positive or negative influence on recruitment or retention. Where a strength-based approach was applied, challenges were framed as opportunities or rewards, while the majority of research questions focused on challenges. Consideration should be given to the approach taken when using research to inform the development of recruitment and retention strategies.

The Model of Retention Equilibrium proposed by Mills and Millsteed [42] weighs incentives to stay in rural practice against incentives to leave. It proves to be a useful framework in considering the interplay of factors that influence therapists’ decision regarding location of practice. This model could assist communities or health service organizations to weigh incentives to stay against incentives to leave and ‘tip the balance in favour of retention’ [42], and it offers a useful means to interpret the findings from the second order analysis. Through providing professional support, professional development and a greater understanding of rural practice, challenges of rural practice (that might be reasons to leave) can be turned into rewards and the incentive to stay.

Despite controversial viewpoints on appraising the quality of qualitative studies, performing an assessment of the studies reviewed in this meta-synthesis offered an additional perspective on the studies’ context and on the variation in quality of the research. In order for qualitative literature to contribute to progressing an issue by changing policy or practice, it is necessary to situate the issue or phenomena within its local context, and link it to the broader literature and phenomena [24]. Overall, the studies reviewed in this meta-synthesis did not clearly define the context in which the research study took place. Without a universal definition for rural [51,52] and given the heterogeneity of rural [53], it is crucial that researchers clearly define the rural context and the nature of rural practice they are researching. Furthermore, as recruitment and retention applies to all stages of careers and sectors of health care (public and private), there is a need to clearly delineate the participant populations so research findings can better inform human resource planning strategies.

The majority of the studies reviewed did not relate their findings to a theory, framework or model, nor did any evaluate the effectiveness of recruitment and retention strategies. Our analysis also found that most strategies were adopted as ‘bundles’ [54] rather than single strategies, which made evaluation difficult. A critical review of the recruitment and retention literature by the World Health Organization suggests that research make better use of theory and frameworks in order to address the multifaceted nature of recruitment and retention factors and to better monitor and evaluate retention interventions [55]. Models such as those presented by Manahan et al. regarding the personal values and experiences of rural health professionals [36] and the Model of Retention Equilibrium [42] provide a framework for situating the complexity of factors and considering the effectiveness of strategies in future studies.

### Limitations

In search of greater insight into factors that influence recruitment and retention of OTs and PTs, this meta-synthesis chose to examine only qualitative literature, a small subset of the recruitment and retention literature. As is the nature of qualitative research, results cannot be easily generalized. However, after establishing the factors common to all studies in this review, this meta-synthesis established first order and second order themes that provide greater understanding of how personal and professional factors contribute to recruitment and retention in rural contexts. While third order analysis has been done in some meta-syntheses, we chose to stop at the second order as there was insufficient depth to the articles we reviewed to achieve further conclusions that would contribute to or progress the literature.

## Conclusions

The current health care environment places considerable emphasis on evidence-informed decisions, and health human resource planning is no exception. This meta-synthesis offers an additional perspective on some of the factors associated with the recruitment and retention of OTs and PTs to rural areas. Of all the factors identified in the original articles and further analysed, professional support from organizations was identified as having a significant effect on recruitment of new graduates and retention across all stages of a career. Opportunities for career development and an understanding of the rural context prior to practising in a rural area were recognized as significant factors in retention. These factors should be prioritized to inform strategies for health human resource planning at all levels of academic and health organizations.

This meta-synthesis also recognizes the need for research to clearly describe the context of rural practice and consider how the positionality of the study (such as a strengths-based inquiry) influences the results. We also recommend the use of a theoretical framework or model to situate recruitment and retention factors in the context of what is known and evaluate the effectiveness of the strategies implemented.

Finding effective and sustainable solutions to the issues of recruitment and retention of rehabilitation professionals to rural and remote areas will ultimately contribute to improving the care and health status of people living in rural communities. To this end, we encourage academic, professional and health care administration communities to invest in professional and organizational support as a part of their recruitment and retention strategy for rehabilitation professionals.

## Competing interests

The authors declare that they do not have any competing interests.

## Authors’ contributions

RKR was responsible for carrying out the systematic literature review, analysis, interpretations and for drafted the manuscript. LCL was involved in reviewing the analysis, providing feedback and editing the manuscript. Both authors read and approved the final manuscript.

## Pre-publication history

The pre-publication history for this paper can be accessed here:

http://www.biomedcentral.com/1472-6963/13/59/prepub
